# Modified thromboelastometric tests provide improved sensitivity and specificity to direct oral anticoagulants compared to standard thromboelastometric tests in-vitro

**DOI:** 10.1186/s12959-022-00400-3

**Published:** 2022-07-21

**Authors:** Philipp Groene, Jennifer Butte, Sarah Thaler, Klaus Görlinger, Simon T. Schäfer

**Affiliations:** 1 Department of Anaesthesiology, University Hospital, LMU Munich, Marchioninistraße 15, 81377 Munich, Germany; 2 TEM Innovations GmbH, Munich, Germany

**Keywords:** Direct-acting oral anticoagulants, Direct factor Xa inhibitors, Thromboelastometry, Direct thrombin inhibitor

## Abstract

**Background:**

The detection of direct oral anticoagulants (DOACs) is still challenging but important in emergency patients. We recently demonstrated that modified thromboelastometry can detect rivaroxaban and dabigatran. Data on the detection rates of modified compared to standard thromboelastometric tests of apixaban and edoxaban, are missing. The aim of this in-vitro dose-effect-study was to add data on these DOACs and to evaluate thromboelastometric tests in-vitro using data of both studies.

**Methods:**

The study was approved by the Ludwig-Maximilians-University ethics committee (No 17-525-2). Written informed consent was obtained from all individuals. Blood samples of healthy volunteers and samples of 10 volunteers for each DOAC were used. Blood samples were spiked with six different concentrations of edoxaban and apixaban (0ng/ml; 31.25ng/ml; 62.5ng/ml; 125ng/ml; 250 ng/ml; 500ng/ml). Modified tests (low-tissue-factor test TFTEM and ecarin-based test ECATEM) as well as standard tests (e.g. FIBTEM) analyzing extrinsic pathway of coagulation were used. Receiver operating characteristics analyzes were performed as well as regression analyzes.

**Results:**

TFTEM CT correlated well with anti-Xa levels of apixaban and edoxaban (apixaban: *r*^2^ = 0.8064 *p* < 0.0001; edoxaban: *r*^2^ = 0.8603; *p* < 0.0001). The detection of direct FXa inhibitors (> 30 ng/mL) was successful with FIBTEM CT with a sensitivity and specificity of 81% and 90%, respectively. As expected, ECATEM CT was not prolonged by direct FXa-inhibitors due to its specificity for direct thrombin inhibitors. Again, TFTEM CT provided the highest sensitivity and specificity for the detection of direct FXa inhibitors with 96% and 95%, respectively. ECATEM test showed 100% sensitivity and 100% specificity for the detection of dabigatran.

**Conclusions:**

Our study presents modified thromboelastometric tests with improved detection of even low DOAC concentrations > 30 ng/mL, including apixaban in-vitro. The study thus complements the previously published data on dabigatran and rivaroxaban. Validation studies must confirm the results due to the explanatory design of this study.

**Supplementary Information:**

The online version contains supplementary material available at 10.1186/s12959-022-00400-3.

## Background

The detection of direct oral anticoagulants (DOACs) is still challenging. Standard coagulation testing such as INR is not helpful and only provides very imprecise and misleading results [1, 2]. Therefore, it is not recommended to monitor DOACs with standard coagulation tests in appropriately anticoagulated patients. Nevertheless, the prompt detection of DOACs is essential in emergency situations and crucial for evidence-based treatment decisions. Examples of such situations are patients with stroke and indication for thrombolysis or patients suffering life-threatening bleeding [3]. In emergency situations, medical history is often not available regarding medication and last intake. Therefore, the timely detection of DOACs and other oral anticoagulants (vitamin K antagonists) is crucial in emergency situations.

Viscoelastic testing is increasingly used and recognized for point-of-care diagnostics in bleeding (risk) management during the last years. Viscoelastometric standard tests are hampered by providing low sensitivity to direct factor Xa inhibitors, particularly apixaban. Therefore, test modifications have been developed and evaluated [4]. The detection of direct thrombin inhibitors (e.g., dabigatran) could be improved by specific tests containing the snake venom ecarin [5–7], as published by our research group [6, 7]. However, the detection of the direct factor Xa inhibitor apixaban is most challenging [4]. We have already been able to show the improved sensitivity of a low-tissue-factor test for the detection of rivaroxaban [7]. The aim of this in-vitro dose-effect-study was to add data on edoxaban and apixaban and to evaluate the possibility of modified compared to standard thromboelastometric tests regarding the detection of DOACs in-vitro, using data of both studies.

## Methods

The study was approved by the Ludwig-Maximilians-University ethics committee (No 17-525-2). Written informed consent was obtained from all individuals prior to study inclusion.

Blood samples of healthy volunteers, 10 in the first study part on rivaroxaban and dabigatran [7] and 20 for the second part with edoxaban and apixaban, were analyzed. Exclusion criteria were coagulation disorders, intake of anticoagulants or platelet inhibitors within 30 days prior to study inclusion. Apixaban/edoxaban were dissolved in dimethyl sulfoxide (DMSO) and aqua to provide stock solution with a concentration of 62.5 ng/µl. Citrated whole blood (S-Monovette Sarstedt, Nürnbrecht, Germany) was spiked with six different concentrations of edoxaban and apixaban (0 ng/ml; 31.25 ng/ml; 62.5 ng/ml; 125 ng/ml; 250 ng/ml; 500 ng/ml). Aqua was added to each sample to ensure that there was no dilutional effect between the samples. DMSO concentration in the blood samples was < 1% and did not affect blood cell viability (> 99%), as assessed using flow cytometry. In preliminary tests, we carried out the specific, calibrated anti-Xa tests to check for correct spiking.

Standard thromboelastometric tests (EXTEM, FIBTEM, HEPTEM, NATEM) were performed using ROTEM *delta* analyzers (TEM Innovations GmbH, Munich, Germany). Additionally, we used modified thromboelastometric tests as described previously by our group: first, a low-tissue-factor test (TFTEM), and second, an ecarin based test (ECATEM).

TFTEM contains about 10% of the tissue factor concentration compared to EXTEM. This makes the assay more sensitive to changes in thrombin generation.

The following parameters provided by the systems were analyzed in the study: clotting time (CT; time from initiation of clotting process to 2 mm clot amplitude), clot formation time (CFT; time from CT until a clot amplitude of 20 mm is reached), and maximum clot firmness (MCF; maximum amplitude of clot firmness).

### Statistics

ROC curve analyzes were done as follows: If a test was to be examined for the detection of a specific DOAC substance with cutoff > 30 ng/ml (e.g., apixaban), all samples spiked with this substance were tested against all samples without a DOAC (control samples without spiking of a DOAC). This enabled us to investigate whether the thromboelastometric tests can distinguish between samples from healthy controls and samples with anticoagulant of the specific substance, even at low doses. To analyze the cutoff > 60 ng/ml the samples with spiked dose of 31.25 ng/ml were added to the controls. This variant makes it possible to investigate the extent to which higher plasma concentrations can be detected, in particular in differentiating between healthy subjects and samples with low DOAC concentrations. We therefore used data of both studies [7]. Optimal cutoff values were determined using the Youden index.

Furthermore, regression analyzes have been performed to calculate coefficient r^2^, slope and intercept.

Data of the edoxaban and apixaban dose-effect-curves are presented as median with interquartile range (Q1/Q3) unless indicated otherwise. The corresponding data on the dose-effect-curves can be found in the first published manuscript for this work [7]. Statistical analysis was performed using Graph Pad Prism 9.2 (Graphpad Software Inc., La Jolla, USA). Statistical differences between groups were analyzed using two-way-ANOVA with post-hoc correction for multiple testing using the Tukey’s multiple comparison test.

Preliminary reference intervals for TFTEM and ECATEM were calculated based on 60 blood samples from healthy individuals without DOAC medication from this and two recently published studies [7, 8].

## Results

Volunteer’s characteristics are displayed in supplemental table 1A. The characteristics of the volunteers for the trials with dabigatran and rivaroxaban can be found in [7]. Any DOAC (> 30 ng/mL) was detected with the FIBTEM CT with a sensitivity of 80% and a specificity of 98%. As expected, ECATEM CT was not able to detect all DOACs (sensitivity of 26% and specificity of 100%) since ECATEM is only sensitive to direct thrombin inhibitors but is not prolonged by direct factor Xa inhibitors. The use of the TFTEM test could significantly increase sensitivity and specificity for DOAC concentrations > 30 ng/mL to 90% and 95%, respectively. For DOAC concentrations > 60 ng/mL, sensitivity and specificity were 88% and 78%, respectively (Table 1).

**Table 1:** Receiver-operating characteristics analyses of the different thromboelastometric tests

Detection Level	ROTEM Variable	AUC	SE	*P*-Value	Sensitivity	Specificity	Optimum cut-off
Apixaban > 30 ng/mL	TFTEM CT	0.9530	0.02285	< 0.0001	88	95	> 179
Apixaban > 30 ng/mL	ECATEM CT	0.6425	0.05871	0.0207	48	85	< 92
Apixaban > 30 ng/mL	TFTEM/ ECATEM CT-ratio	0.9455	0.02458	< 0.0001	84	95	> 1.988
Apixaban > 30 ng/mL	EXTEM CT	0.8965	0.03687	< 0.001	70	95	> 68
Apixaban > 30 ng/mL	FIBTEM CT	0.7935	0.04646	< 0.0001	68	78	> 66
Apixaban > 30 ng/mL	HEPTEM CT	0.7740	0.06150	0.0004	76	75	> 195
Apixaban > 30 ng/mL	NATEM CT	0.6000	0.06037	0.1044	80	40	> 779
Apixaban > 60 ng/mL	TFTEM CT	0.9605	0.02503	< 0.0001	93	94	> 210
Apixaban > 60 ng/mL	ECATEM CT	0.6343	0.05876	0.0292	55	72	< 95
Apixaban > 60 ng/mL	EXTEM CT	0.9236	0.03171	< 0.0001	83	90	> 68
Apixaban > 60 ng/mL	FIBTEM CT	0.8588	0.04026	< 0.0001	80	78	> 66
Apixaban > 60 ng/mL	HEPTEM CT	0.7975	0.05315	< 0.0001	67	49	> 195
Apixaban > 60 ng/mL	NATEM CT	0.6458	0.05851	0.0179	65	60	> 858
Apixaban > 60 ng/ml	TFTEM/ ECATEM CT-ratio	0.9570	0.02555	< 0.0001	85	98	> 2.460
Edoxaban > 30 ng/mL	TFTEM CT	0.9975	0.002884	< 0.0001	100	98	> 199
Edoxaban > 30 ng/mL	ECATEM CT	0.6520	0.05802	0.0136	52	70	< 96
Edoxaban > 30 ng/mL	TFTEM/ ECATEM CT-ratio	0.9985	0.001759	< 0.0001	96	100	> 2.244
Edoxaban > 30 ng/mL	EXTEM CT	0.9990	0.001691	< 0.0001	98	100	> 73
Edoxaban > 30 ng/mL	FIBTEM CT	0.9738	0.01303	< 0.0001	94	90	> 72
Edoxaban > 30 ng/mL	HEPTEM CT	0.9455	0.02461	< 0.0001	82	95	> 213
Edoxaban > 30 ng/mL	NATEM CT	0.6525	0.05782	0.0133	46	90	> 1041
Edoxaban > 60 ng/mL	TFTEM CT	0.9870	0.007937	< 0.0001	98	92	> 264
Edoxaban > 60 ng/mL	ECATEM CT	0.5710	0.06042	0.2489	80	34	< 101
Edoxaban > 60 ng/mL	EXTEM CT	0.9896	0.008586	< 0.0001	95	100	> 84
Edoxaban > 60 ng/mL	FIBTEM CT	0.9905	0.007477	< 0.0001	95	98	> 81
Edoxaban > 60 ng/mL	HEPTEM CT	0.9358	0.02911	< 0.0001	85	97	> 222
Edoxaban > 60 ng/mL	NATEM CT	0.7330	0.05592	0.0002	55	90	> 1041
Edoxaban > 60 ng/mL	TFTEM/ ECATEM CT-ratio	0.9765	0.01263	< 0.0001	98	90	> 2.719
Rivaroxaban > 30 ng/mL	TFTEM CT	0.9980	0.002176	< 0.0001	98	98	> 198
Rivaroxaban > 30 ng/mL	ECATEM CT	0.6585	0.05850	0.0101	62	73	< 103
Rivaroxaban > 30 ng/mL	TFTEM/ ECATEM CT-ratio	0.9975	0.002879	< 0.0001	98	100	> 2.180
Rivaroxaban > 30 ng/mL	FIBTEM CT	0.9985	0.001892	< 0.0001	98	100	> 86
Rivaroxaban > 30 ng/mL	NATEM CT	0.8125	0.04512	< 0.0001	84	68	> 896
Rivaroxaban > 60 ng/mL	TFTEM CT	0.9850	0.008727	< 0.0001	93	96	> 304
Rivaroxaban > 60 ng/mL	ECATEM CT	0.7060	0.05722	0.0008	73	70	> 101
Rivaroxaban > 60 ng/mL	FIBTEM CT	0.9890	0.007630	< 0.0001	93	98	> 106
Rivaroxaban > 60 ng/mL	NATEM CT	0.8425	0.04153	< 0.0001	78	80	> 953
Rivaroxaban > 60 ng/mL	TFTEM/ ECATEM CT-ratio	0.9770	0.01268	< 0.0001	98	90	> 2.568
Any DXaI > 30 ng/mL	TFTEM CT	0.9828	0.008352	< 0.0001	96	95	> 179
Any DXaI > 30 ng/mL	ECATEM CT	0.5453	0.04771	0.3788	39	78	< 95
Any DXaI > 30 ng/mL	TFTEM/ ECATEM CT-ratio	0.9805	0.008711	< 0.0001	90	100	> 2.180
Any DXaI > 30 ng/mL	FIBTEM CT	0.9219	0.01925	< 0.0001	81	90	> 72
Any DXaI > 30 ng/mL	NATEM CT	0.6883	0.04377	0.0003	52	80	> 948
Any DXaI > 60 ng/mL	TFTEM CT	0.9494	0.01469	< 0.0001	89	86	> 264
Any DXaI > 60 ng/mL	ECATEM CT	0.5214	0.04320	0.6235	25	84	> 107
Any DXaI > 60 ng/mL	TFTEM/ ECATEM CT-ratio	0.9387	0.01728	< 0.0001	90	86	> 2.719
Any DXaI > 60 ng/mL	FIBTEM CT	0.8971	0.02208	< 0.0001	81	86	> 79
Any DXaI > 60 ng/mL	NATEM CT	0.7362	0.03582	< 0.0001	61	81	> 948
Dabigatran > 30 ng/mL	TFTEM CT	0.9460	0.02185	< 0.0001	96	83	> 150
Dabigatran > 30 ng/mL	ECATEM CT	1.0	0.0	< 0.0001	100	100	> 128
Dabigatran > 30 ng/mL	TFTEM/ ECATEM CT-ratio	0.9190	0.02917	< 0.0001	78	100	< 0.9251
Dabigatran > 30 ng/mL	FIBTEM CT	0.9975	0.002520	< 0.0001	98	98	> 78
Dabigatran > 30 ng/mL	NATEM CT	0.9820	0.01001	< 0.0001	96	90	> 1049
Dabigatran > 60 ng/mL	TFTEM CT	0.9578	0.01826	< 0.0001	95	84	> 164
Dabigatran > 60 ng/mL	ECATEM CT	1.0	0.0	< 0.0001	100	100	> 177
Dabigatran > 60 ng/mL	FIBTEM CT	0.9823	0.01004	< 0.0001	95	92	> 97
Dabigatran > 60 ng/mL	NATEM CT	0.9760	0.01212	< 0.0001	93	90	> 1182
Dabigatran > 60 ng/mL	TFTEM/ ECATEM CT-ratio	0.9420	0.02975	< 0.0001	88	100	< 0.8779
Any DOAC > 30 ng/mL	TFTEM CT	0.9736	0.009820	< 0.001	90	95	> 179
Any DOAC > 30 ng/mL	ECATEM CT	0.5910	0.03951	0.0693	26	100	> 123
Any DOAC > 30 ng/mL	FIBTEM CT	0.9408	0.01481	< 0.0001	80	98	> 78
Any DOAC > 30 ng/mL	NATEM CT	0.7618	0.03522	< 0.0001	55	90	> 1041
Any DOAC > 30 ng/ml	TFTEM/ECATEM ratio	0.7556	0.02939	< 0.0001	68	100	> 2.180
Any DOAC > 60 ng/mL	TFTEM CT	0.9200	0.01693	< 0.0001	88	78	> 219
Any DOAC > 60 ng/mL	ECATEM CT	0.5921	0.03689	0.0200	25	100	> 177
Any DOAC > 60 ng/mL	FIBTEM CT	0.8958	0.01974	< 0.0001	86	78	> 79
Any DOAC > 60 ng/mL	NATEM CT	0.7563	0.03090	< 0.0001	62	81	> 1041
Any DOAC > 60 ng/ml	TFTEM/ECATEM ratio	0.7186	0.03370	< 0.0001	68	88	> 2.719

*AUC* Area under the curve, *SE* Standard error, *CT* Clotting time, *DOAC* Direct oral anticoagulant

The detection of direct FXa inhibitors (> 30 ng/mL) was successful with FIBTEM CT with a sensitivity and specificity of 81% and 90%, respectively. As expected, ECATEM CT was not prolonged by direct FXa inhibitors due to its specificity for direct thrombin inhibitors (sensitivity of 39% and specificity of 78%). Again, TFTEM CT provided the highest sensitivity and specificity for the detection of direct FXa inhibitors with 96% and 95%, respectively. Using the ratio between TFTEM CT and ECATEM CT, as presented in our first publication on rivaroxaban and dabigatran, showed a high sensitivity of 90% and specificity of 100% to direct FXa inhibitors, as well. Sensitivity and specificity to detect direct FXa inhibitor concentrations > 60 ng/mL are displayed in Table 1.

Sensitivity and specificity to detect each single DOAC were analyzed, too. FIBTEM CT already showed a good sensitivity and specificity for the detection of edoxaban (94% and 90%, respectively), rivaroxaban (98% and 100%, respectively), and dabigatran (98% and 98%, respectively) at whole blood concentrations of > 30 ng/mL. For apixaban sensitivity and specificity were only 68% and 78%, respectively. HEPTEM CT, representing the intrinsic pathway of coagulation, was less sensitive and specific to direct FXa inhibitors compared to FIBTEM CT (edoxaban: 82% and 95%, apixaban: 67% and49%, respectively). In contrast, TFTEM CT detected all direct FXa inhibitors with a sensitivity and specificity for apixaban of 88% and 95%, respectively, for edoxaban of 88% and 95%, respectively, and for rivaroxaban of 100% and 98%, respectively. TFTEM CT also provided a high sensitivity and specificity for dabigatran (96% and 83%, respectively). The use of the ratio between TFTEM CT and ECATEM CT improved detection of all FXa inhibitors compared to FIBTEM test (apixaban: 84% and 95%; edoxaban: 96% and 100%, and rivaroxaban: 98% and 100%, respectively). ECATEM test showed 100% sensitivity and 100% specificity for the detection of dabigatran regardless of the selected whole blood concentration (> 30 ng/mL or > 60 ng/mL. Further data are displayed in Table 1.

**Fig. 1:**
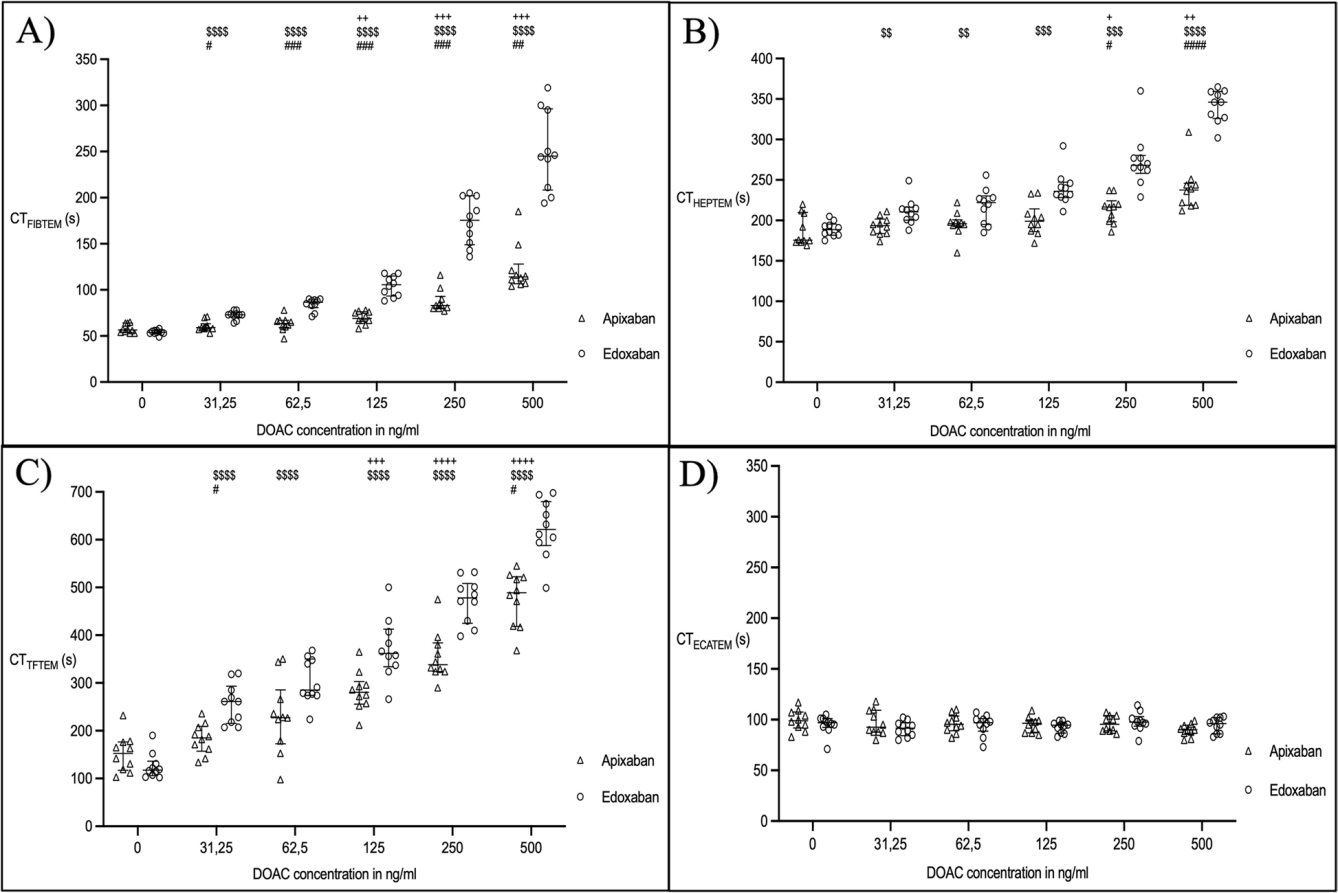
Clotting time in the different thromboelastometric tests after spiking blood samples with edoxaban and apixaban. Depicted are (**A**) FIBTEM, (**B**) HEPTEM, (**C**) TFTEM and (**D**) ECATEM. Data presented as median and interquartile range. + versus baseline apixaban; $ versus baseline edoxaban; # between DOACs at this concentration. CT: clotting time; DOAC: direct oral anticoagulant; FIBTEM: analyzing the extrinsic pathway of coagulation focusing on fibrinogen status; HEPTEM: analyzing the intrinsic pathway of coagulation containing heparinase; TFTEM: low tissue factor test containing 10% of tissue factor compared to standard EXTEM/FIBTEM; ECATEM: ecarin-based test for the specific detection of direct thrombin inhibitors

Based on the first part of the study, we also created dose-effect-curves for edoxaban and apixaban (Fig. 1). In the FIBTEM test, CT was prolonged by both edoxaban and apixaban, with edoxaban showing the stronger effect on CT (Fig. 1). Only edoxaban resulted in a significant prolongation of FIBTEM CT at low whole blood concentrations (baseline: 54 s (53/56) vs. 31.25 ng/mL: 73 s (71/75); *p* < 0.0001). In case of apixaban, a significant increase was only seen at concentrations of 125 ng/mL (baseline: 57 s (54/64) vs. 125 ng/mL: 69 s (66/76); *p* < 0.0001). The same applies for EXTEM CT. In HEPTEM test, CT was only prolonged by apixaban at concentrations of > 250 ng/mL (baseline: 176 s (173/210) vs. 250 ng/mL: 217 s (198/224); *p* = 0.0327). Here again, edoxaban led to a significant CT prolongation already at low concentrations of 31.25 ng/mL (baseline: 189 s (182/196) vs. 31.25 ng/mL: 211 s (200/216); *p* = 0.0033). In the TFTEM test, CT was significantly prolonged by apixaban from a concentration of 125 ng/mL (baseline: 152 s (117/177) vs. 125 ng/mL: 281 s (256/303); *p* = 0.003). Even if it was not statistically significant, the TFTEM test already showed a clearly noticeable CT prolongation at low concentrations compared to the FIBTEM and EXTEM test. In case of edoxaban, CT prolongation was seen again at 31.25 ng/mL (baseline: 118 s (106/136) vs. 31.25 ng/mL: 261 s (215/293); *p* < 0.0001). The ECATEM test showed no significant CT prolongation by either of the two DOAKs. Further data are presented in Table 2.

**Table 2:** Thromboelastometric results of the different tests with edoxaban and apixaban. Data are presented as median and (Q1/Q3)

	Apixaban						Edoxaban					
	**0** **ng/ml**	**31.25 ng/ml**	**62.5 ng/ml**	**125 ng/ml**	**250 ng/ml**	**500 ng/ml**	**0 ng/ml**	**31.25 ng/ml**	**62.5 ng/ml**	**125 ng/ml**	**250 ng/ml**	**500 ng/ml**
**CT**_**NATEM**_	802 (678/875)	799 (701/882)	802 (761/955)	824 (694/925)	1006 (824/1110)	1017 (905/1085)	756 (574/875)	802 (586/886)	795 (719/969)	894 (737/1199)	1212 (995/1338)	1231 (1108/1405)
**CT** ^**EXTEM**^	56 (52/63)	61 (59/67)	66 (61/72)	72 (69/75)	88 (81/97)	115 (98/129)	57 (56/62)	79 (76/80)	86 (84/90)	108 (102/120)	175 (148/202)	255 (215/284)
**CT**_**FIBTEM**_	57 (54/64)	59 (58/63)	64 (59/67)	69 (66/76)	83 (80/93)	114 (107/128)	54 (53/56)	73 (71/75)	87 (81/89)	106 (93/115)	176 (149/202)	245 (208/296)
**CT**_**HEPTEM**_	176 (173/210)	194 (184/202)	196 (192/201)	199 (186/214)	217 (198/224)	238 (219/246)	189 (182/196)	211 (200/216)	222 (195/230)	236 (228/247)	268 (258/280)	346 (326/359)
**CT**_**TFTEM**_	152 (117/177)	185 (157/209)	228 (173/286)	281 (256/303)	338 (324/386)	489 (419/522)	118 (106/136)	261 (215/293)	285 (274/350)	362 (334/413)	478 (425/509)	621 (588/680)
**CT**_**ECATEM**_	99 (92/108)	93 (88/109)	96 (89/104)	97 (87/100)	96 (89/104)	90 (86/95)	97 (92/101)	92 (84/97)	97 (89/101)	95 (87/97)	98 (94/103)	96 (86/102)

*CT* Clotting time

Furthermore, we evaluated the correlation between CT results and whole blood concentrations of the two DOACs as reported for rivaroxaban and dabigatran recently [7]. Results are displayed in supplemental Table 2. Preliminary reference intervals of the TFTEM and ECATEM tests are shown in Table 3.

**Table 3:** Preliminary Reference Intervals (Median + 95% CI). Data based on samples without DOAC spiking

ROTEM test	N	CT [s]	CFT [s]	Alpha [˚]	A5 [mm]	A10 [mm]	A20 [mm]	MCF [mm]	LI30 [%]	maxV [mm/min]	maxV-t [s]	AUC [mm x min]
**TFTEM**	60	129 (119–135)	92 (84–104)	72 (70–74)	43 (41–44)	55 (53–56)	62 (59–63)	63 (61–64)	100 (100–100)	14 (12–15)	154 (148–175)	6219 (6110–6442)
**ECATEM**	60	97 (93–100)	79 (75–84)	74 (73–75)	47 (46–49)	58 (56–59)	64 (62–66)	64 (63–67)	100 (100–100)	17 (16–18)	136 (132–141)	6412 (6237–6671)

*CT *Clotting time, *CFT *Clot formation time, *MCF: A5/10/20* amplitude 5/10/20min after, *CT* maximum clot firmness; *LI30* Lysis index after 30 min; *AUC* Area under the curve

## Discussion

The results of our study show that the modified thromboelastometric tests TFTEM and ECATEM are more sensitive and specific in the detection of the DOACs compared to standard viscoelastic tests in-vitro explanatory study. Using the low tissue factor test TFTEM improved detection of all FXa inhibitors, particularly of apixaban. The ecarin based ECATEM test is specific for direct thrombin inhibitors as previously shown [7] and was not altered by rivaroxaban, edoxaban and apixaban.

An improved detection of even low DOAC concentrations > 30 ng/mL is of great clinical interest and importance. A rapid evaluation of the coagulation status must be carried out not only in bleeding patients but also in patients before thrombolysis after ischemic stroke, especially in situations where the patients cannot communicate anymore with the physician [3]. To date there have been attempts to solve this problem of real-time detection with thromboelastometry [4, 9]. However, it turned out that standard thromboelastometric tests are not detecting low apixaban concentrations, reliably. Modified thromboelastometric tests showed promising results with improved detection rates in-vitro, here. The ecarin-based test is highly specific for direct thrombin inhibitors such as dabigatran, since ECATEM CT is not prolonged by direct FXa inhibitors [5–7]. The development of the low-tissue factor tests already showed improved detectability for rivaroxaban [7, 10, 11]. A thromboelastometry test based on Russel-Viper-Venom (RVV-test) in a recently published study provided similar sensitivity and specificity to apixaban concentrations > 50 ng/mL (ROC AUC of 0.8974, sensitivity of 80%, and specificity of 88%) compared to EXTEM CT for apixaban concentration > 30 ng/mL [6, 12]. However, detection of low apixaban blood concentrations > 30 ng/mL seems to be superior to both, RVV-test as well as EXTEM and FIBTEM using the TFTEM test. Nevertheless, it can be seen that the low tissue factor test also shows marginal overlaps in the low concentration range. The determined cutoff of 179s represents an optimal combination of sensitivity and specificity. An improved sensitivity would be at the expense of specificity and vice versa. The lower sensitivity and specificity to detect DOAC concentrations > 60 ng/mL compared to > 30 ng/mL can be explained by pooling data with 0 ng/mL and 31.25 ng/mL in these ROC analyzes. Accordingly, the tests had even to discriminate between blood concentrations of 31.25 ng/mL and 62.5 ng/mL in these analyses which was still possible with high sensitivity and specificity. The quantification of DOAC concentrations when the DOAC was known, could be confirmed for apixaban and edoxaban by regression analysis as done for rivaroxaban and dabigatran in our previous study [7].

Accordingly, the use of these modified thromboelastometric tests may be helpful in the future to detect patients under DOAC therapy in real-time, and therefore, may allow for guiding specific therapy with antidots such as andexanet alfa or idarucizumab in bleeding patients treated with DOACs. Of course, these tests have to be validated in further clinical trials. Here, the use of thromboelastometric test combinations in conjunction with decision-tree analysis showed already promising results [8].

Our study has several limitations: First, spiked blood samples have been used in this in-vitro study. This approach was crucial for conducting dose-effect-curves and to evaluate the effects of the DOACs over a large concentration range although the effect of in-vitro added anticoagulants may be greater than that of anticoagulants in ex-vivo samples. Consequently, follow-up studies to evaluate ex-vivo samples from clinical patients are planned to verify and validate our results (DETECT-study, trial registration number: DRKS00028569). The ROC analyzes carried out, do not contain any other aspects from real samples that could influence the detection, such as dilution effects, and therefore may overestimate the detection rates in absolute terms.

## Conclusions

In summary, our study presents modified thromboelastometric tests with improved detection of even low DOAC concentrations > 30 ng/mL, including apixaban compared to standard thromboelastometric tests in-vitro. The study thus complements the previously published data on dabigatran and rivaroxaban. Due to the experimental approach and the explanatory design, the tests have to be examined in further studies using real patient samples to approve the promising results of this study.

## Supplementary information

**Additional file 1:** **SupplementalTable 1. **Volunteer characteristics. Values are median (Q1/Q3)or number (proportion).

**Additional file 2:** **Supplemental Table 2. **Correlation between ROTEM test CT results and Apixaban and Edoxaban whole bloodconcentrations.

## Data Availability

The datasets used and/or analyzed during the current study are available from the corresponding author on reasonable request.

## References

[1] Gosselin RC, Adcock DM, Douxfils J (2019). An update on laboratory assessment for direct oral anticoagulants (DOACs). Int J Lab Hematol.

[2] Samuelson BT, Cuker A, Siegal DM, Crowther M, Garcia DA (2017). Laboratory Assessment of the Anticoagulant Activity of Direct Oral Anticoagulants: A Systematic Review. Chest.

[3] Berge E, Whiteley W, Audebert H, De Marchis GM, Fonseca AC, Padiglioni C (2021). European Stroke Organisation (ESO) guidelines on intravenous thrombolysis for acute ischaemic stroke. Eur Stroke J.

[4] Seyve L, Richarme C, Polack B, Marlu R (2018). Impact of four direct oral anticoagulants on rotational thromboelastometry (ROTEM). Int J Lab Hematol.

[5] Körber MK, Langer E, Köhr M, Wernecke K-D, Korte W, von Heymann C (2017). In vitro and ex vivo Measurement of Prophylactic Dabigatran Concentrations with a New Ecarin-Based Thromboelastometry Test. Transfus Med Hemother. Karger Publishers.

[6] Groene P, Wagner D, Kammerer T, Kellert L, Giebl A, Massberg S (2021). Viscoelastometry for detecting oral anticoagulants. Thromb J.

[7] Schäfer ST, Wiederkehr T, Kammerer T, Acevedo A-C, Feil K, Kellert L (2020). Real-time detection and differentiation of direct oral anticoagulants (rivaroxaban and dabigatran) using modified thromboelastometric reagents. Thromb Res.

[8] Schäfer ST, Otto A-C, Acevedo A-C, Görlinger K, Massberg S, Kammerer T (2021). Point-of-care detection and differentiation of anticoagulant therapy - development of thromboelastometry-guided decision-making support algorithms. Thromb J.

[9] Henskens YMC, Gulpen AJW, van Oerle R, Wetzels R, Verhezen P, Spronk H (2018). Detecting clinically relevant rivaroxaban or dabigatran levels by routine coagulation tests or thromboelastography in a cohort of patients with atrial fibrillation. Thromb J.

[10] Pailleret C, Jourdi G, Siguret V, Gouin-Thibault I, Gandrille S, Stepanian A (2019). Modified ROTEM for the detection of rivaroxaban and apixaban anticoagulant activity in whole blood: A diagnostic test study. Eur J Anaesthesiol.

[11] Adelmann D, Wiegele M, Wohlgemuth RK, Koch S, Frantal S, Quehenberger P (2014). Measuring the activity of apixaban and rivaroxaban with rotational thrombelastometry. Thromb Res. Elsevier.

[12] Oberladstätter D, Voelckel W, Schlimp C, Zipperle J, Ziegler B, Grottke O (2021). A prospective observational study of the rapid detection of clinically-relevant plasma direct oral anticoagulant levels following acute traumatic injury. Anaesthesia..

