# Supplementary material for: Modified thromboelastometric tests provide improved sensitivity and specificity to direct oral anticoagulants compared to standard thromboelastometric tests in-vitro
Source: Thromb J. 2022 Jul 21;20:40. doi: 10.1186/s12959-022-00400-3 (PMC9306144; doi:10.1186/s12959-022-00400-3)
Supplement: Supplementary file 1 — Additional file 1: SupplementalTable 1. Volunteer characteristics. Values are median (Q1/Q3)or number (proportion). [file 12959_2022_400_MOESM1_ESM.docx]

# **Supplemental Table 1: Volunteer characteristics.** Values are median (Q1/Q3) or number (proportion).

| Healthy volunteers | | |
| --- | --- | --- |
|  | Apixaban  (n=10) | Edoxaban  (n=10) |
| **Gender** (women/men) | 5/5  (50/50) | 5/5  (50/50) |
| **Age** (years) | 30  (24-34) | 31  (27-33) |
| **Body mass index** (kg*m^2^) | 23.3  (21.9-26.3) | 22.6  (21.2-23.1) |
| **Former diseases** (yes/no) | 5/5  (50/50) | 4/60  (40/60) |
| **Medication intake** (yes/ no) | 4/60  (40/60) | 4/6  (40/60) |
| **Haemoglobin** (g/dl) | 15.5  (13.7-15.9) | 14.6  (14.2-15.7) |
| **Haematocrit** (%) | 0.44  (0.40-0.45) | 0.42  (0.41-0.45) |
| **Platelets** (10^9^/L) | 248  (233-302) | 247  (196-272) |
| **INR** | 0.9  (0.9-1.0) | 1.0  (0.9-1.0) |
| **Quick** (%) | 112  (108-117) | 107  (102-114) |
| **aPTT** (s) | 26  (24-26) | 25  (25-26) |
| **Thrombin time** (s) | 17  (17-18) | 18  (18-18) |
| **Fibrinogen** (mg/dl) | 291  (268-346) | 237  (220-270) |
| **Creatinine** (mg/dl) | 0.85  (0.7-1.0) | 0.8  (0.8-0.9) |
| **GFR** (ml/min) | 108  (90-121) | 111  (96-118) |

INR: international normalized ratio; aPTT: activated partial thromboplastin times; GFR: glomerular filtration rate

Underlying diseases: n=5 Hypothyroidism, n=1 aortic stenosis, n=1 arterial hypertension, n=1 multiple sclerosis, n=1 chronic sinusitis.

Medication intake: n=5 levothyroxin, n=1 glataramiracetat, n=1 Budesonid, n=1 olmesartan, n=1 amlodipin.
