# Supplementary material for: Modified thromboelastometric tests provide improved sensitivity and specificity to direct oral anticoagulants compared to standard thromboelastometric tests in-vitro
Source: Thromb J. 2022 Jul 21;20:40. doi: 10.1186/s12959-022-00400-3 (PMC9306144; doi:10.1186/s12959-022-00400-3)
Supplement: Supplementary file 2 — Additional file 2: Supplemental Table 2. Correlation between ROTEM test CT results and Apixaban and Edoxaban whole bloodconcentrations. [file 12959_2022_400_MOESM2_ESM.docx]

**Supplemental table 2: Correlation between ROTEM test CT results and Apixaban and Edoxaban whole blood concentrations**

| **DOAC** | **ROTEM test CT** | **Correlation Coefficient r^2^** | **Slope** | **Intercept** | **P-value** |
| --- | --- | --- | --- | --- | --- |
| **Apixaban** | TFTEM | 0.8064 | 0.5998 | 205.9 | <0.0001 |
|  | EXTEM | 0.8072 | 0.1176 | 61.5 | <0.0001 |
|  | FIBTEM | 0.7731 | 0.1349 | 58.29 | <0.0001 |
|  | HEPTEM | 0.4820 | 0.1002 | 191.8 | <0.0001 |
| **Edoxaban** | TFTEM | 0.8603 | 1.166 | 263.4 | <0.0001 |
|  | EXTEM | 0.9002 | 0.5841 | 69.03 | <0.0001 |
|  | FIBTEM | 0.9005 | 0.5978 | 63.98 | <0.0001 |
|  | HEPTEM | 0.8282 | 0.4310 | 204.3 | <0.0001 |

CT: clotting time
